# St. George's Hospital

**Published:** 1906-07-07

**Authors:** 


### The Hospital

A Journal of

The Medical Sciences and Hospital Administration.

Vol. XL.—No. 1,033. SATURDAY, JULY 7 1906.

### St. George's Hospital.

The special meeting of the Governors of St. George's Hospital which we reported in full last week presents in the result arrived at an amazing contrast to the stir and movement of public opinion outside. The pendulum has swung so far in the direction of advanced Liberal opinions that numbers of people regard the result of the last election as proving that the country is within measurable distance of upheaval, socialism, and the most radical changes in the British constitution itself. Hyde Park Corner occupies a central position in the metropolis of the Empire, and the board-room of St. George's Hospital stands upon this central site. Yet a visitor to the special Court of Governors on the 26th ultimo, listening to the discussion following the able speech of the Treasurer, Mr. West, might have felt from the attitude taken up by the majority of the speakers, that he was a Rip Van Winkle, who had fallen asleep in Noah's Ark, and after many centuries had woke up to find the opinions of his shipmates in that memorable craft held and supported by the people of London, as represented by the majority of the Governors present at the meeting. Listening with astonishment to the view that the best progress was to stand still, some enlightenment might gradually have come to him from the statements of various speakers that they had been governors for fifty years, and that they were well contented to let things remain as they were.

The present deadlock at St. George's Hospital is an apt illustration of the dangers of an open Board, which may kill all active interest on the part of the younger men. Without an infusion of new blood families and institutions must die out and fail. At St. George's a number of worthy men have grown old with the institution; they have naturally become attached to the old condition of affairs, have persuaded themselves that it cannot be improved upon, and, being left much to themselves, have come to regard any change as one to be looked upon as suspect, and the introduction of an energetic, up-to-date, businesslike administration as something to be resisted and defeated at all costs. This is the explanation of the circumstance that an able, businesslike, exhaustive speech by Mr. West, the Treasurer, in which he clearly indicated and proved by figures that the best interests of the hospital called for one line of action and one only, should have been disregarded and set aside in favour of a resolution to continue the habit of the older men of marking time whilst the funds grow less, and the reputation of the whole institution and its well-being and future credit are imperilled.

Fortunately public opinion is now fully alive to the relative needs and claims of the greater hospitals of London. No big hospital can become modernised, nor can it indeed exist on the old lines, without it secures the goodwill and the material support of the larger givers to the voluntary hospitals. Now these givers for the most part are men of business; the King's and other Funds have enabled them to keep in touch with the actual circumstances of the greater hospitals; they are fully aware of the only course which can succeed in regard to St. George's Hospital, and until that course is followed money will no doubt be withheld from the institution, and it must remain *in statu quo*, with a tendency to retrograde by losing a number of its supporters and being each year shorter and shorter of funds. So the proceedings at the special meeting to which we have referred have in fact little importance, except to illustrate the hopelessness of attempting to make St. George's into an up-to-date modern hospital whilst its affairs are controlled by a small minority of unprogressive spirits who have rendered good service in the past, but whose views are altogether out of sympathy with hospital needs in the present day. St. George's Hospital will, no doubt, in due course sell its present site and migrate to a district where the population urgently need the hospital care which it can afford. Mr. West's scheme, like the report of the sub-committee which sat for over six months, is based upon business principles. It entails the sale of the site, which Mr. West has good reason to believe can be made to realise £400,000, and, if he is right, the adoption of this scheme should obviate the necessity of an appeal to the public This being so, the profession and the public will agree that the proper course for St. George's Hospital to adopt is to put its house in order by the adoption of business methods and securing a new hospital on an adequate site with all reasonable despatch, whereby the permanent income of the hospital must be materially increased.

